# Inclusion of climate change strategies in municipal Integrated Development Plans: A case from seven municipalities in Limpopo Province, South Africa

**DOI:** 10.4102/jamba.v8i3.245

**Published:** 2016-04-26

**Authors:** Mankolo X. Lethoko

**Affiliations:** 1 Turfloop Graduate School of Leadership, University of Limpopo, South Africa

## Abstract

The Intergovernmental Panel on Climate Change (IPCC) has made it clear that anthropogenic greenhouse gasses are the main cause of observed global warming that leads to climate change. Climate change is now a global reality. In the South African political set-up, local municipalities are the structures that are in direct contact with communities and they draw up Integrated Development Plans (IDPs), which are reviewed and upgraded annually. The article seeks to investigate the extent to which climate change adaptation and mitigation strategies are embedded IDPs in seven vulnerable municipalities in the Limpopo Province. The article conducted an in-depth content analysis of the IDPs of the seven municipalities and the results have revealed that these municipalities have not included adaptation and mitigation strategies adequately in their IDPs despite being the most vulnerable municipalities in the province. The article concludes that these municipalities have not as yet institutionalised climate change in their daily operations, planning and decision making. To this end, the paper recommends that local municipalities should include climate change adaptation and mitigation strategies in their IDPs.

## Introduction and background

According to the Intergovernmental Panel on Climate Change (2013):

a large part of global warming is irreversible: from the point where emissions have dropped to zero, global temperature will remain almost constant for centuries at the elevated level reached by that time. (p. 6)

Mukheibir and Ziervogel (2007:147) maintain that stakeholders should be engaged in order to identify vulnerable sectors and existing and potential climate change adaptation and mitigation initiatives. Climate change mitigation and adaptation have been the subject of discussion in many countries as the impact of climate change is experienced at the local level by communities. Thus, local governance systems are often ‘closest’ entities for planning and implementing both the adaptation and mitigation strategies suitable for a particular geographical and social context in which they are located (Pasquini, Cowling & Ziervogel 2013:225). Within the South African context, local governance refers to municipalities. The *Municipal Systems Act* (No. 32 of 2000), chapter 5, part 1, section 25, outlines that all municipalities are mandated to develop Integrated Development Plans (IDPs) (Department of Provincial and Local government 2000a). The IDP is the main planning document for any local municipality in the country. Therefore, the vulnerability of community from climate change depends on the policies and frameworks that the local municipality puts in place through the IDP.

This article has utilised content analysis as a research methodology in an attempt to do an in-depth analysis of the content of IDPs for Limpopo’s seven most vulnerable municipalities in terms of climate change. The article examines the inclusion of climate change adaptation and mitigation strategies in the IDPs. The work is anchored on the principle that it is the responsibility of local municipalities to protect, assist and guide communities on how to adapt and mitigate the effects of climate change on their livelihood and sustainable development. Laukkonen *et al*. (2009) point out that:

the vulnerability of individuals and communities to climate change impact is not simply determined by the location of their settlements, but also how these settlements are serviced, how effective and capable their local governments are and to what extend communities are able to cope with climate change impact. (p. 287)

A sole objective was set for investigation as: to determine the extent of the inclusion of climate change adaptation and mitigation strategies in the IDP of the seven vulnerable municipalities in Limpopo, South Africa. The next section provides a brief overview of the theoretical framework that informs the article and research methodology used in the article.

## Research methodology and theoretical framework

This article has used an in-depth content analysis of the IDPs for Limpopo’s seven most vulnerable municipalities in terms of climate change. The author did a page by page analysis of the 2013/2014 IDPs of the following vulnerable municipalities: Greater Giyani, Ephraim Mogale, Greater Letaba, Thulamela, Aganang, Fetakgomo and Elias Motsoaledi. Welman, Kruger and Mitchell (2005:221) define content analysis as ‘a qualitative analysis of qualitative data’. The basic technique involves counting the frequencies and sequencing of particular words, phrases and concepts in order to identify keywords and themes. Babbie and Mouton (2012) define the same concept as:

[*a*] research method which examines words or phrases within a wide range of texts (IDPs in this case) including books, book chapters, essays, interviews, speeches and informal conversations. By examining the presence of repetition of certain words and phrases in these texts, a researcher is able to make inferences about the philosophical assumptions of a writer, a written piece, the audience to which the piece was written and even the culture and the time in which the text is embedded. (p. 491)

The South African Local Government Association *Let’s Respond Guide and Toolkit* (Department of Environmental Affairs 2012) and Municipal Adaptation Plans were utilised as rich sources of information. The next section discusses South Africa’s participation in climate change arena internationally and also the national efforts made by the government in as far as climate change is concerned including the legislative frameworks for climate change in South Africa.

This article’s theoretical framework is based on three concepts, namely the vulnerability of municipalities towards climate change, the adaptive capacity of communities to the impact of climate change and the mitigation strategies which should be included in the municipal IDPs. It is through the IDP that a municipality plans and budgets for climate change impact. Vulnerability is identified as a core concept in disaster risk reduction, livelihood and the quest for poverty eradication (Miller *et al*. 2010:11). The Intergovernmental Panel on Climate Change (2007:6) defines vulnerability as the degree to which geophysical, biological and socio-economic systems are susceptible to and unable to cope with the adverse impact of climate change.

According to Kasperson and Dow (2001), the world has been experiencing:

a worsening trend of human suffering and economic losses from natural disasters over the past several decades. In the last 40 years, the number of ‘great disasters’ has increased by factor of four whilst economic losses have increased by a factor of 10. The significance of these events to the social vulnerability of exposed human populations is of special concern. (p. 147)

In addition, Laukkonen *et al*. (2009:287) purport that the vulnerability of individuals and communities to climate change impact is not simply determined by the location of their settlements but also how these settlements are serviced, how effective and capable their local governments are and to what extent communities are able to cope with climate change impact. This implies that local municipalities as ‘settlements’ where communities live have to be effective and well serviced in order to assist communities in their struggle to adapt and mitigate climate change. However, if a municipality does not have a clear plan through an IDP of how they intend to adapt and mitigate the effects of climate change, then the communities’ adaptive capacity is adversely affected.

The United Nations Development Programme (UNDP) points out that the science is clear and the debate is over as climate change is happening and there is a need to act now without delay. The route now and the most common strategies are to cope and/or learn to live with the impact of climate change (adaptation) and to reduce the impact of climate change (mitigation) (United Nations Development Programme 2008:41). It is within this scenario of adaptation and mitigation that municipalities have to assist communities to adapt to and mitigate the effects of climate change. Table 1 shows the difference between adaptation and mitigation as part of the conceptual framework of the article.

**TABLE 1:** Difference between adaptation and mitigation.

Adaptation	Mitigation
Most common strategies aimed to cope with the impact of climate change	Most common strategies to reduce with the impact of climate change
A focus on adaptation now is sometimes perceived as an acknowledgement of allocating scarce resources to a threat that is not yet perceived as imminent	Includes technical and infrastructural investments in order to reduce the impact of climate change. e.g. turning off lights in empty rooms
Requires long-term strategies that may not be popular with government systems which prefer to focus on shorter time frames and quick fixes	Mitigation is a global responsibility, e.g. limiting greenhouse emissions is a global responsibility because the benefits are global, not necessarily regional
Can be more localised and region specific where specific realities of climate change occur	Is global and not localised

*Source*: Author adopted from Laukkonen, J., Blanco, K.P., Lenhart, J., Keiner, M., Cavric, B. & Kinuthia-Njenga, C., 2009, ‘Combining climate change and mitigation measures at the local level’, *Habitat International* 33, 287–292

It emerges therefore that adaptation and mitigation strategies are supportive of each other and are interlinked. Hence, incorporating both strategies to reduce or cope with climate change is no longer a ‘nice to have’ for municipalities but inevitable as climate change is a reality. In essence, climate change will almost certainly make the process of eradicating poverty and to achieve the incoming Sustainable Development Goals more difficult (United Nations Millennium project 2012). This is because of its direct effect on poor people’s livelihoods and assets upon which they depend on like land and water resources that are slowly depleting as a result of the impact of climate change. It is against this backdrop that the article seeks to investigate the inclusion of climate change adaptation and mitigation strategies in IDPs in the seven vulnerable municipalities in Limpopo Province of South Africa.

### Addressing climate change in South Africa

South Africa has been an active participant in various fora and conventions that deal with climate change since 1994. The country has shown great commitment to sustainable development through both international participation and national frameworks. These frameworks include some of the following: the Kyoto Protocol, the United Nations Framework Convention on Climate Change, the Cancun Agreement, the Convention on International Trade in Endangered Species of Wild Fauna and Flora, the Ramsah Convention on Wetlands of International Importance, the Basel Convention on the Control of Transboundary Movement of Hazardous Wastes and Their Disposal, and the Montreal Protocol for the Protection of the Ozone Layer (Montmasson-Clair 2012:56). This implies that South Africa’s participation in climate change within the international arena is outstanding. This is also supported by the numerous policies, guidelines and legislative frameworks that have been promulgated. Table 2 provides an overview of these policies and legal frameworks and summarises their relevant goals.

**TABLE 2:** Selected climate change policies and their main goals in South Africa.

Policies and measures	Main goals
Framework for Environmental Fiscal Reform (National Treasury 2006)	Provides principles and guidelines for fair and effective environmental taxes
Ten Year Innovation Plan (Department of Science and Technology 2008)	Includes safe, clean, affordable and reliable energy supply and climate change as priorities
National Framework for Sustainable Development (2008)	Supports sustainable development initiatives
Medium-Term Strategic Framework 2009–2014 (NPC 2009)	Notes the need for sustainable livelihoods and sustainable resource management and relates these to various other policy areas including energy, water, housing, technology and competitiveness
Industrial Policy Action Plan (the Department of Trade and Industry 2010, 2011 and 2012)	Specifically targets growth in green industries, focusing on not only solar water heaters (SWHs) but also wind energy, biofuels, electric vehicles and organic farming
New Growth Path (EDD 2010)	Targets the growth of a green economy, resulting in 400 000 new and additional jobs
National Climate Change Response Green Paper (2010)	Makes a fair contribution to the global effort to achieve the stabilisation of greenhouse gas concentrations in the atmosphere, adapt to and manage unavoidable and potential damaging climate change impacts through interventions that built and sustain South Africa’s social, economic and environmental resilience
Green Economy Accord (2010)	Has 12 commitments related to the green economy
Integrated Resource Plan 2010–2030 (Department of Education 2011)	Limits emissions from electricity generation to 275 Mt per year, expects renewable energy to make up 42% of all new electricity generation the next 20 years
National Climate Change Response White Paper (Republic South Africa 2011)	Endorses and quantifies South Africa’s greenhouse gas emissions limits/commitments, aims to grow green jobs whilst limiting job loss in unsustainable industries
National Strategy for Sustainable Development (Department of Environmental Affairs 2011)	A large variety of indicators and goals spanning social, economic and environmental issues, but no budgets, timelines or responsibilities
National Development Plan Vision 2030 (NDP) 2011	The NDP is very specific about its goals and focuses on energy and carbon

*Source*: Adapted from Montmasson-Clair, G., 2012, *Green economy policy framework and employment opportunity: A South African case article*, Trade and Industrial Policy Strategies Publishers, Pretoria, pp. 6–9

From Table 2, it emerges that South Africa has successfully managed to establish various legal frameworks for climate change, including sector-specific legislation in waste management, carbon tax, transport, energy efficiency, renewable energy and others.

Integrated Development Planning (IDP) is a process through which municipalities prepare a strategic plan containing short-, medium- and long-term development objectives, strategies and programmes for the municipal area. The IDP is a principal instrument that guides and informs budgeting, management and decision making related to service delivery and development in a municipality (Department of Provincial and Local Government 2000b). The IDP process enables municipalities to work together with communities and other stakeholders to find innovative and cost-effective ways of eradicating poverty and growing the local economy.

In addition, from the National Climate Change Response White Paper (Department of Environmental Affairs 2011), one of its strategic priorities is linked to the IDP. The White Paper asserts that the government will:

prioritise the mainstreaming of climate change considerations and responses into all relevant sector, national, provincial and local planning regimes such as but not limited to, the Industrial Policy Action Plan, Integrated Resource Plan for Electricity Generation, Provincial Growth and Development Plans and Integrated Development Plans. (p. 14)

This implies that the IDP is the most relevant document that can assist municipalities in their planning and budgeting for climate change. However, in the article as it will be discussed later, the seven vulnerable municipalities in the Limpopo Province do not have clear and well-spelt adaptation and mitigation strategies for climate change in their municipalities. The national government has been involved in various local government initiatives in order to enhance South African municipalities’ understanding of climate change and these initiatives are discussed later.

In 2008, the Department of Science and Technology (DST) adopted a Ten Year Innovation Plan aimed at moving the country towards a new knowledge economy. The Ten Year Plan is underpinned by five grand challenges, one of which is Science and Technology for Global change with an emphasis on climate change (Department of Science and Technology 2007). The DST has also established a Bureau of Global Change Science that includes the South African Environmental Observation Network (SAEON), Africa Centre for Climate and Earth Systems Science (ACCESS), Africa Earth Observatory Network (AEON), The South African National Space Agency (SANSA) and South African Risk and Vulnerability Atlas (SARVA) (Department of Science and Technology 2015).

From the SARVA (Department of Science and Technology 2015), the Minister of Science and Technology asserts that:

Africa is one of the most vulnerable continents to climate variability and change, a situation aggravated by the interaction of ‘multiple stresses’, occurring at various levels, and low adaptive capacity. (p. 21)

The South African Risk and Vulnerability Atlas is a flagship science-into-policy initiative of the Department of Science and Technology’s Global Change Grand Challenge (Department of Science and Technology 2015). The SARVA’s purpose is to ensure that decision makers in local municipalities have relevant information on the impact and risks associated with climate change in Africa and South Africa in particular. The Atlas provides easily understood global change sensitivity and vulnerability information at regional, national, provincial and municipal levels for policy makers and decision makers at all levels for everyone who wants to know more about global change and its impacts on South Africa (Department of Science and Technology 2015). From the Department of Environmental Affairs, South African Local Government Association (SALGA) and Department of Co-operative Governance (Department of Environmental Affairs 2012), The *Let’s Respond Guide and Toolkit* has been designed to achieve a number of objects (Box 1).

**BOX 1:** Objects of let’s respond.

To take municipalities and their leaders (elected and corporate) through necessary steps towards climate-responsive planning and provides a set of tools to support the process.
To assist municipalities to identify communities and sectors that might be at risk from the impact of climate change and variability and explore opportunities.
To assist municipalities in the prioritisation of local government response actions.
To support the integration of these priorities into IDP and related municipal sector plans.
To align climate response with existing climate and development challenges and deepen existing response capacity.
To develop links with research institutions and community bodies to improve the flow of information such as early warning systems.
To improve cross-sector integration of management and development planning.
To move more efficiently on core development objectives, providing both short-term to long-term climate response benefits.

*Source*: Department of Environmental Affairs, 2012, *Let’s respond: An introduction to integrating climate change risks and opportunities into municipal planning*, Government Printers, Pretoria

According to Roberts (2008:527), the extent to which an issue such as climate change becomes successfully institutionalised in day-to-day operations, planning and decision making at the local level by using institutional markers is outlined as follows. There is the emergence of an identifiable political or administrative champion(s) for climate change issues. These champions will assist the municipalities to ensure that climate change issues are involved and taken care of in all municipal operations. Then comes the appearance of climate change as a significant issue in mainstream municipal plans such as the IDP, Integrated Waste Management Plan, Environmental Plan, Spatial Plan and many more. This will be followed by the allocation of dedicated resources (human and financial) to climate change issues. Lastly, there will be a need to incorporate climate change considerations into political and administrative decision making.

The Ethekwini municipality is identified as having been able to develop its own Municipal Climate Protection Programme. According to the municipality’s website (www.durban.gov.za), in 2004 Ethekwini Municipality initiated a Municipal Climate Protection Programme (MCPP). An important intervention included in the MCPP is the mainstreaming of climate change considerations into all aspects of the work undertaken by the municipality. This structure has ensured that climate change issues are not just linked to environmental matters but even in fields such as supply chain, marketing and many more.

Pather-Elias (2011) defines a municipal adaptation plan as a strategy that aims to assist the municipalities to cope with the impact of a changing climate and implement strategies to ensure sustainability of livelihoods and natural resources in order to build resilience and adapt. The plan should be integrated into the new and existing plans and should align with other regional plans, for example IDP. Mukheibir and Ziervogel (2007:146) have listed the following 10 steps on how to design a Municipal Climate Change Adaptation Plan:

- Assess current climate trends and future projections for the geographical region.
- Undertake a climate vulnerability assessment of the municipal area.
- Review current development plans and priorities, for example IDP, Spatial Plan, etc.
- Overlay and overlap development priorities, expected climate change, current climate vulnerability and expected future climate vulnerability using GIS for spatial interrogation.
- Develop adaptation options using new and existing tools.
- Prioritise the adaptation actions using tools such as multi-criteria analysis (MCA), cost–benefit analysis (CBA) or social accounting matrices (SAM).
- Develop programme and project scoping and design documents together with associated budget. This document will be the Municipal Adaptation Plan.
- Implement the interventions prioritised by MAP.
- Monitor and evaluate the interventions on an on-going basis.
- Regularly review and modify the plan at predefined intervals.

## Discussion

### Findings from the study

The author studied and analysed the content of IDPs for seven municipalities deemed most vulnerable in the Limpopo Province of South Africa. The focus was to assess how the said municipalities were mainstreaming climate change adaptation and mitigation strategies in their IDPs. Table 3 illustrates the extent to which the IDPs selected from the seven most vulnerable municipalities in the province included climate change information.

**TABLE 3:** Inclusion of climate change information in Integrated Development Plans.

Name of the municipality	IDP year	Climate change content
1. Greater Giyani	2013–2014	Environmental challenges which include pollution, water pollution, deforestation, veld and forest fires, soil erosion, informal settlements and overgrazing
2. Greater Letaba	2013–2014	Environmental challenges which include pollution, water pollution, deforestation, veld and forest fires, soil erosion, informal settlements and alien plant invaders, drought and natural disasters Integrated Waste Management Plan Environmental capacity-building Environmental SWOT analysis
3. Thulamela	2013–2014	Environmental challenges which include water supply, water sources, water conservation and demand, water backlogs and challenges, climate, climate change, environmental and natural resource management, air quality, hydrology/water resources, waste management and waste disposal sites
4. Aganang	2013–2014	Environmental management plan which was prepared in 2009 Integrated Waste Management Plan - status quo report prepared in 2004
5. Fetakgomo	2013–2014	Environmental challenges such as rainfall patterns, El Nino and La Nina susceptibility, air quality, uncontrolled fires, biodiversity, soil erosion, over-utilisation of land, environmental management, water and sanitation
6. Elias Motsoaledi	2013–2014	Environmental challenges such as water, water backlogs, sanitation, waste management, establishment of a unit, shallow discussions on climate change, rainfall, temperature, environmental conservation and sensitive areas, air quality and pollution, climate change, soil erosion, wetlands, emissions/air pollution, water pollution, deforestation, awareness and by-laws
7. Ephraim Mogale	2013–2014	Environmental challenges such as water scarcity, water pollution, air quality and surface pollution

IDP, Integrated Development Plans; SWOT, Strength, Weakness, Opportunity, Threat.

Having studied and analysed the seven IDPs from the selected municipalities, it became clear that there are similarities and differences amongst them. The following main differences were observed from the analysis. The Fetakgomo municipality is aware of its vulnerable status and this was mentioned in the IDP. The Aganang and Greater Letaba municipalities were the only municipalities that had an Integrated Waste Management Plan and an Environmental Management Plan, respectively. However, these were old, requiring face-lifting to address modern climate change challenges.

The similarities were also noticeable and included the following: all the seven IDPs studied listed climate change challenges without proposals on how to adapt and mitigate against those challenges. Six municipalities did not even mention their vulnerability status in the IDP, although this is crucial. Water scarcity was highlighted as a generic challenge for all the municipalities. This is so as the Limpopo Province is a water-scarce province. Furthermore, all the municipalities included pollution as one of their environmental challenges. Some of them referred to it as air quality, whilst others included water and surface pollution, especially in Ephraim Mogale municipality. Waste management was also cited as a problem in all municipalities except Ephraim Mogale.

The general observation was that all the IDPs did not have a structured way of presenting their environmental plans in the IDPs. All of them only listed the problems in their various municipalities with very little discussion relating to the strategies to adapt and mitigate the existing problems especially linked to their vulnerability status. This is a challenge that requires further investigation in terms of providing capacity and related solutions for the municipalities. The next section cusses on the emerging suggestions to enhance climate change adaptation and mitigation mainstreaming in the IDPs for the sample municipalities.

### Suggestions to enhance climate change mainstreaming in Integrated Development Plans

Based on findings presented herein, for the seven municipalities, the study would like to propose the following suggestions for enhancing the mainstreaming of climate change adaptation and mitigation in the IDPs. There is a need for the national government to assist local municipalities, especially the seven most vulnerable in the Limpopo Province with training and up-skilling opportunities so that they can be able to implement existing policies and guidelines on climate change. Local municipality officials and politicians need to familiarise themselves with the vulnerability status of their own municipalities so that their IDPs reflect their adaptation and mitigation plans. The government’s *Let’s Respond Toolkit*, being implemented in partnership with the South African Local Government Association and other stakeholders and partners has come up with the process to assist municipalities into how to integrate climate into IDPs. Therefore, the sample municipalities are encouraged to seek assistance and join this initiative to adopt the guidelines.

The *Let’s Respond Toolkit* has been implemented successfully in other pilot municipalities, including Amathole District Municipality of the Eastern Cape Province (Department of Environmental Affairs 2011). Lessons learnt from this implementation are valuable for benchmarking in the sample seven municipalities in the Limpopo Province. The *Let’s Respond Toolkit* and campaign embraces the following process that municipalities should embrace in mainstreaming climate change into the IDP processes (Department of Environmental Affairs 2011):

- Preparation – The lead department in a municipality is mandated to drive climate change integration and establish an interdepartmental climate change response committee.
- Analysis – climate change challenges are identified and assessed.
- Strategy – the development of municipal climate change vision and strategy is done and climate change priorities are integrated into the IDP.
- Projects – climate change priority projects are detailed and reviewed to align them to climate change dimensions.
- Integration and Implementation – IDP programmes meet climate-responsive criteria (Department of Environmental Affairs 2011).

## Conclusion

When I looked at the IDPs from the seven most vulnerable municipalities from the Limpopo Province of South Africa, a number of challenges that these municipalities were experiencing in addressing climate change surfaced. Amongst such challenges could be listed: low local human capacity to undertake this kind of planning; limited knowledge and understanding of climate issues at local level; limited financial resources and competing resources which often result in medium- to long-term planning being side-lined; projects that do not fit into the short political life of decision makers are not implemented. It is difficult to convince decision makers to consider a need for a climate strategy when climate projections cover a longer time horizon than political and development agendas of municipalities. This implies that there is a need for an extensive engagement process to bring politicians and decision makers on board in terms of climate adaptation and mitigation. However, the reality is that scientific evidence confirms that climate change impact can already be felt in various spheres of people’s lives all over the world and that most of the warming observed in the past is because of human activities. This situation implies that societies and municipalities have to make plans to adapt and mitigate the impact of climate change before it is too late.
